# A survey into the current fitness testing practices of elite male soccer practitioners: from assessment to communicating results

**DOI:** 10.3389/fphys.2024.1376047

**Published:** 2024-03-19

**Authors:** Nikolaos D. Asimakidis, Chris J. Bishop, Marco Beato, Irvin N. Mukandi, Adam L. Kelly, Anthony Weldon, Anthony N. Turner

**Affiliations:** ^1^ Faculty of Science and Technology, London Sport Institute, Middlesex University, London, United Kingdom; ^2^ School of Health and Sports Sciences, University of Suffolk, Ipswich, United Kingdom; ^3^ Faculty of Health, Education and Life Sciences, Birmingham City University, Birmingham, United Kingdom

**Keywords:** football, professional, data analysis, data reporting, physical performance, assessment

## Abstract

This study provides insight into the current fitness testing practices in elite male soccer. One hundred and two practitioners from professional soccer leagues across 24 countries completed an online survey comprising 29 questions, with five sections: a) background information, b) testing selection, c) testing implementation, d) data analysis, and e) data reporting. Frequency analysis was used to evaluate the responses to fixed response questions and thematic analysis was used for open-ended questions to generate clear and distinct themes. Strength (85%) and aerobic capacity (82%) represent the most frequently assessed physical qualities. Scientific literature (80%) is the most influential factor in testing selection and practitioners conduct fitness testing less frequently than their perceived ideal frequency per season (3.6 ± 2 vs. 4.5 ± 2). Time and competitive schedule were the greatest barriers to fitness testing administration. Practitioners mostly used a ‘hybrid’ approach (45%) to fitness testing, blending ‘traditional’ (i.e., a day dedicated to testing) and ‘integrated’ (i.e., testing within regular training sessions) methods. Microsoft Excel is the most used software for data analysis (95%) and visualization (79%). An equal use of the combination of best and mean scores of multiple trials (44%) and the best score (42%) was reported. Comparing a player’s test performance with previous scores (89%) was the most common method for interpreting test results. However, only 38% considered measurement error. Digital displays and verbal feedback are the most common data reporting methods, with different data reporting processes for coaches and players. Practitioners can use data and findings from this study to inform their current testing practices and researchers to further identify areas for investigation, with the overarching aim of developing the field of fitness testing in elite male soccer.

## Introduction

Soccer is a sport where success depends on technical, tactical, physical, and psychological factors (Turner and Stewart, 2014). Accordingly, soccer match-play requires players to execute different high-intensity activities, such as kicking, tackling, turning, jumping, and sprinting (Stølen et al., 2005). The physical demands are ever-increasing (Bush et al., 2015; Zhou et al., 2020), with elite soccer players covering distances of ∼14 km per game, with ∼10% being at speeds >19.8 km/h (Dolci et al., 2020). The number of matches played by professional players has also increased, with a soccer season in the English Premier League and other major European soccer leagues comprising ∼60 matches, including domestic, cup, and international competitions (Nassis et al., 2020). A high level of athleticism seems to be essential to cope with the demands of the modern game. For example, research has shown differences in physical attributes (e.g., speed, power, strength, change of direction ability, and aerobic capacity) between starting vs. non-starting players (Hoppe et al., 2020), senior vs. youth (Cardoso De Araújo et al., 2018), and elite vs. non-elite players (Kobal et al., 2016). Therefore, fitness testing is necessary to provide practitioners (e.g., strength and conditioning [S&C] coaches, sports scientists) with objective information on the physical capacity of individuals and teams, which can be used to benchmark players, design and evaluate individualized training programs, reduce injury risk, inform return-to-play processes, and contribute to talent identification (Turner et al., 2011; Taylor et al., 2022; Weakley et al., 2023).

Employing a comprehensive fitness testing battery supports the development of well-rounded and physically robust soccer players (Svensson and Drust, 2005; Beato et al., 2023). Therefore, testing selection should be based on general and position-specific requirements of soccer (e.g., biomechanical and physiological aspects) (Turner et al., 2011), and ‘testing for the sake of testing’ should be avoided if it does not provide value to the training process. Practitioners face the challenge of choosing between various assessments and outcome variables, since a standardized universal testing battery in soccer has yet to be established. Typically, soccer fitness testing batteries include aerobic capacity, linear speed, strength, power, reactive strength, change of direction (COD), and repeated sprint ability (RSA) tests (Turner et al., 2011; Taylor et al., 2022). To navigate the uncertainty of the testing selection process, practitioners must select tests that measure the intended capacity (validity) and ensure results are representative of the athlete’s ability (reliability) (McGuigan, 2016; Weakley et al., 2023). Furthermore, tests should be sensitive enough to detect small but meaningful changes in performance to demonstrate players’ physical progress (Paul and Nassis, 2015). Accordingly, external factors such as equipment availability, number of athletes, age and training status of athletes, competitive schedule, time efficiency, simplicity, practicality, and timing can influence the testing selection and administration process (McGuigan et al., 2013).

The selected fitness testing battery will likely result in vast amounts of data requiring in-depth analysis for teams and individual players (Turner et al., 2021; Turner, 2022). Therefore, practitioners require advanced data analysis skills to distinguish the ‘signal’ from the ‘noise’ (Hopkins, 2000). This will enable them to interpret and present results effectively to other members of the athlete support team (e.g., coaches) and inform the wider training process (Buchheit, 2017). Therefore, practitioners should aim to create intuitive and informative reports, as coaches may not possess a statistical background (Thornton et al., 2019). As players are in the middle of the data collection and reporting process, results must be clearly communicated to them and actioned accordingly, thus leading to increased buy-in. Nevertheless, there is no ‘one size fits all’ when reporting test results, and the audience’s preference will determine the output.

Although research has extensively examined different methods to measure fitness in elite soccer players, limited evidence exists on how the ‘science’ of fitness testing is translated into practice, particularly concerning testing data analysis and reporting. Previous survey-based research provided insight into fitness testing in soccer; however, this formed part of a larger survey instrument and did not directly ask about data analysis and reporting (Beere and Jeffreys, 2021; Weldon et al., 2021), or the sample consisted of mixed backgrounds (elite and non-elite, male and female) (McQuilliam et al., 2023). Therefore, this survey study aims to acquire real-world insights into the fitness testing processes being conducted in elite male soccer. Our results will provide valuable information on the selection, implementation, data analysis, and data reporting of fitness tests, providing a basis of information for practitioners and researchers. Furthermore, potential areas for further investigation and research will also be highlighted.

## Materials and methods

### Participants

Participants were recruited using a digital invitation via the research team’s network and use of online platforms (i.e., X [formerly Twitter] and LinkedIn). Chain sampling was used to maximize the sample size, whereby practitioners were requested to share the survey with their elite soccer network. The inclusion criteria to ensure that collected responses represented the current testing practices in elite soccer required participants to be involved in a professional soccer club working with male players >17 years old. Participants gave their consent by clicking the relevant box on the introductory page of the survey. All participants were ≥18 years old. ***REMOVED FOR PEER REVIEW*** research and ethics committee at ***REMOVED FOR PEER REVIEW*** provided ethical approval for the study.

### Study design

The online survey platform SurveyMonkey (San Mateo, California, United States) was used to create and host the survey. Initially, the survey underwent pilot testing with three S&C coaches working in elite male soccer; two with a PhD and one with a master’s degree, all with more than 7 years’ of experience in elite soccer, and three researchers with an applied soccer background and more than 9 years in academia, to assess content validity. This led to minor modifications and rewording of questions to ensure they were clear and appropriate for the intended population. The introductory page of the survey outlined the purpose of the study, general information, confidentiality of information, and included the informed consent statement. The survey lasted ∼15 min, contained five sections: 1) background information, 2) testing selection, 3) testing implementation, 4) data analysis, and 5) data reporting), and included a combination of fixed response and open-ended questions (see Supplementary Appendix S1). Some questions allowed more than one answer, resulting in some questions having more answers than others.

### Data acquisition and statistical analyses

All responses from SurveyMonkey were exported into a customized Excel spreadsheet (Microsoft Corporation, Redmond, Washington, United States) for further analysis. The data collection period was from the first of July 2023 to the 15th of December 2023. Data were analysed and presented using a range of descriptive statistics, including the calculation of the mean, standard deviation, absolute frequencies (counts), and relative frequencies (percentages). A frequency analysis was undertaken with fixed response questions. Open-ended questions were evaluated using a thematic analysis approach (Braun and Clarke, 2019), similar to previous survey studies in elite soccer (Weldon et al., 2021; Loturco et al., 2022). This thematic analysis approach consisted of the subsequent six-step framework: 1) familiarization with the data, 2) generating initial codes, 3) searching for themes, 4) reviewing themes, 5) defining and naming themes, and 6) producing the report. The key themes arising from the raw responses were generated for each open-ended question by the lead author and agreed upon by all co-authors with extensive experience physically testing athletes.

## Results

### Demographics

One hundred and two elite male soccer practitioners, consisting of 32 S&C coaches, 27 physical performance coaches, 24 sports scientists, nine directors/heads of performance, seven physiotherapists, and three technical coaches, with professional experience of 8.2 ± 5.7 years, took part in this study. Practitioners worked in professional soccer across 24 countries, including Italy (28.4%), The United Kingdom (24.5%), Germany (7.8%), The United States of America and Portugal (each 4.9%), Spain (3.9%), Greece (2.9%), Australia, Cyprus, Denmark, India, Scotland and Sweden (each 1.9%), and Belgium, Bosnia and Herzegovina, Croatia, France, Georgia, Ireland, Netherlands, Northern Ireland, Saudi Arabia, Singapore and Turkey (each 1%). Regarding academic background, 63% had a master’s degree, 18% had a PhD degree, 15% had a bachelor’s degree, and 4% were PhD candidates. Professional qualifications were widely held by respondents, including soccer coaching licenses (62.7%), National Strength and Conditioning Association (NSCA) Certified Strength and Conditioning Specialist (CSCS) (22.5%), United Kingdom Strength and Conditioning Association (UKSCA) Accredited Strength and Conditioning Coach (ASCC) (8.8%), British Association of Sport and Exercise Sciences (BASES) Accredited Sport and Exercise Scientist (7.1%), and NSCA Certified Performance and Sport Scientist (CPSS) (2.9%).

### Testing selection

**FIGURE 1:**
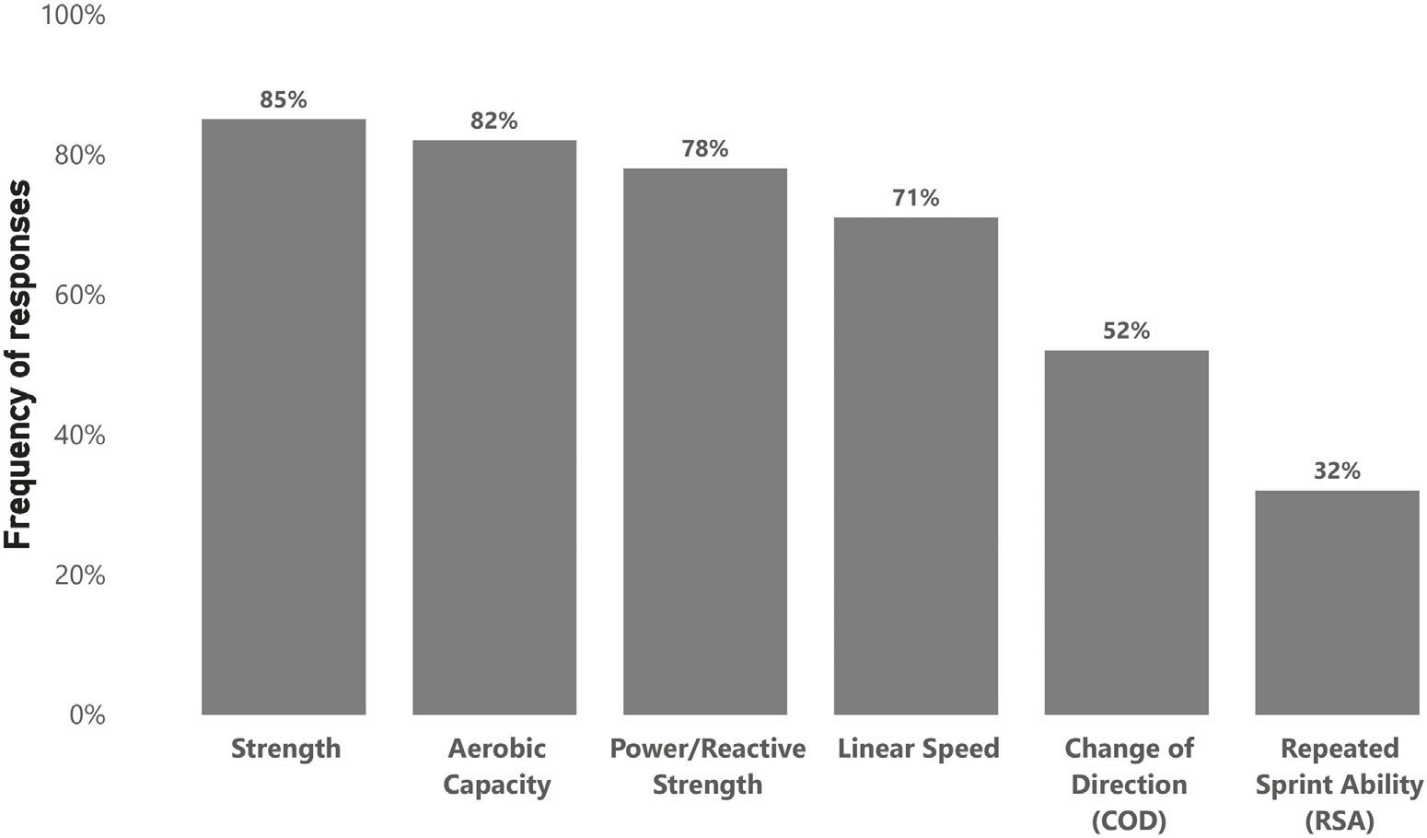
Physical capacities that practitioners assess (n = 102).

Figure 1 illustrates the frequency of responses regarding the physical capacities that practitioners assessed within their fitness testing batteries. Table 1 represents the most common tests used to assess each physical capacity. For the factors influencing the selection of testing methods, 83 participants (81%) responded. The most common responses were published scientific literature (80%), constraints (e.g., time, budget, equipment) (60%), expert opinion or professional experience (59%), needs analysis of the sport (58%), specific needs or goals of the team or players (40%), usefulness of a test (established from in-house test-retest reliability) (35%), prescribed from national governing bodies (10%), and ‘other’ reasons (7%) (e.g., the ability of a test to inform programming, the ability to inform the return-to-play process, and the historical use of tests in the club).

**TABLE 1:** Most common tests reported by practitioners to assess each physical quality.

Physical capacity (number of respondents)	Test	Percentage of respondents
Strength (n = 79)	Isometric Mid-Thigh Pull (IMTP)	28/79 (35%)
	Isometric Adductor Strength (Groin Squeeze Test)	27/79 (34%)
	Isokinetic Strength of the Quadriceps and/or Hamstrings	23/79 (29%)
	3RM Squat	18/79 (23%)
	3RM Bench Press	13/79 (16%)
	Other: Nordic Hamstring Strength Test, Isometric Hamstring Strength Test, Max Pull-Ups, 3RM Pull-Up, Isometric Calf Raise, 1RM Trap Bar Deadlift	13/79 (16%)
	1RM Squat	12/79 (15%)
	Predicted 1RM using Barbell Velocity	10/79 (13%)
	1RM Bench Press	8/79 (10%)
	Isometric Squat	7/79 (9%)
	Flywheel Testing	4/79 (5%)
Aerobic Capacity (n = 78)	30–15 Intermittent Fitness Test	23/78 (29%)
	Yo-Yo Intermittent Recovery Test 2	19/78 (24%)
	Yo-Yo Intermittent Recovery Test 1	17/78 (22%)
	Specified Distance for Time	17/78 (22%)
	Incremental Treadmill Test to Exhaustion	12/78 (15%)
	Submaximal Test	10/78 (13%)
	Specified Time for Distance	9/78 (12%)
	Multi-stage Fitness Test (Beep Test)	7/78 (9%)
	VAMEVAL Test	4/78 (5%)
	Other: Mognoni’s Test, Bosco Test	3/78 (4%)
	University of Montreal Track test	1/78 (1%)
Power/Reactive Strength (n = 74)	Countermovement Jump (CMJ)	68/74 (92%)
	Squat Jump (SJ)	33/74 (45%)
	Single-leg Countermovement Jump (SL CMJ)	32/74 (43%)
	Drop Jump (DJ)	28/74 (38%)
	10/5 Repeated Jumps Test	18/74 (24%)
	Triple Hop Test	15/74 (20%)
	Single-leg Hop Test	14/74 (19%)
	Single-leg Drop Jump	12/74 (16%)
	Other: Standing Broad Jump, Triple Broad Jump, Trap Bar Squat Jump	8/74 (11%)
	Vertical Jump with Free Arms	7/74 (9%)
Linear Speed (n = 67)	10 m	43/67 (64%)
	30 m	39/67 (58%)
	20 m	32/67 (48%)
	5 m	28/67 (42%)
	40 m	11/67 (16%)
	20 m Flying	8/67 (12%)
	30 m Flying	7/67 (10%)
	Other: 50m, 60m, Max Velocity with GPS	5/67 (7%)
	10 m Flying	4/67 (6%)
	40 m Flying	4/67 (6%)
	5 m Flying	0/67 (0%)
COD (n = 49)	505 Test	27/49 (55%)
	*t*-Test	9/49 (18%)
	Illinois Agility Test	8/49 (16%)
	Other: COD Test, Pro Agility Test, Modified 505 Test, In-house COD Test (10 + 10 m with 90° Cut)	8/49 (16%)
	Arrowhead Agility Test	6/49 (12%)
	Zig-Zag Test	0/49 (0%)
RSA (n = 30)	7 × 30 m Sprint with 20s Rest	10/30 (33%)
	6 × 40 m Sprint with 20s Rest	7/30 (23%)
	8 × 30 m Sprint with 25s Active Recovery	6/30 (20%)
	6 × 40 m (20 + 20 m with 180° Turns) Shuttle Sprint with 20s Rest	6/30 (20%)
	Other: 6 × 35 m Sprint with 10s Rest, 6 × 25 m Sprint with 25s Rest, 5 × 30 m with 20s Active Recovery	5/30 (17%)

^a^ RM: repetition maximum.

^a^ GPS: global positioning system.

### Testing implementation

**FIGURE 2:**
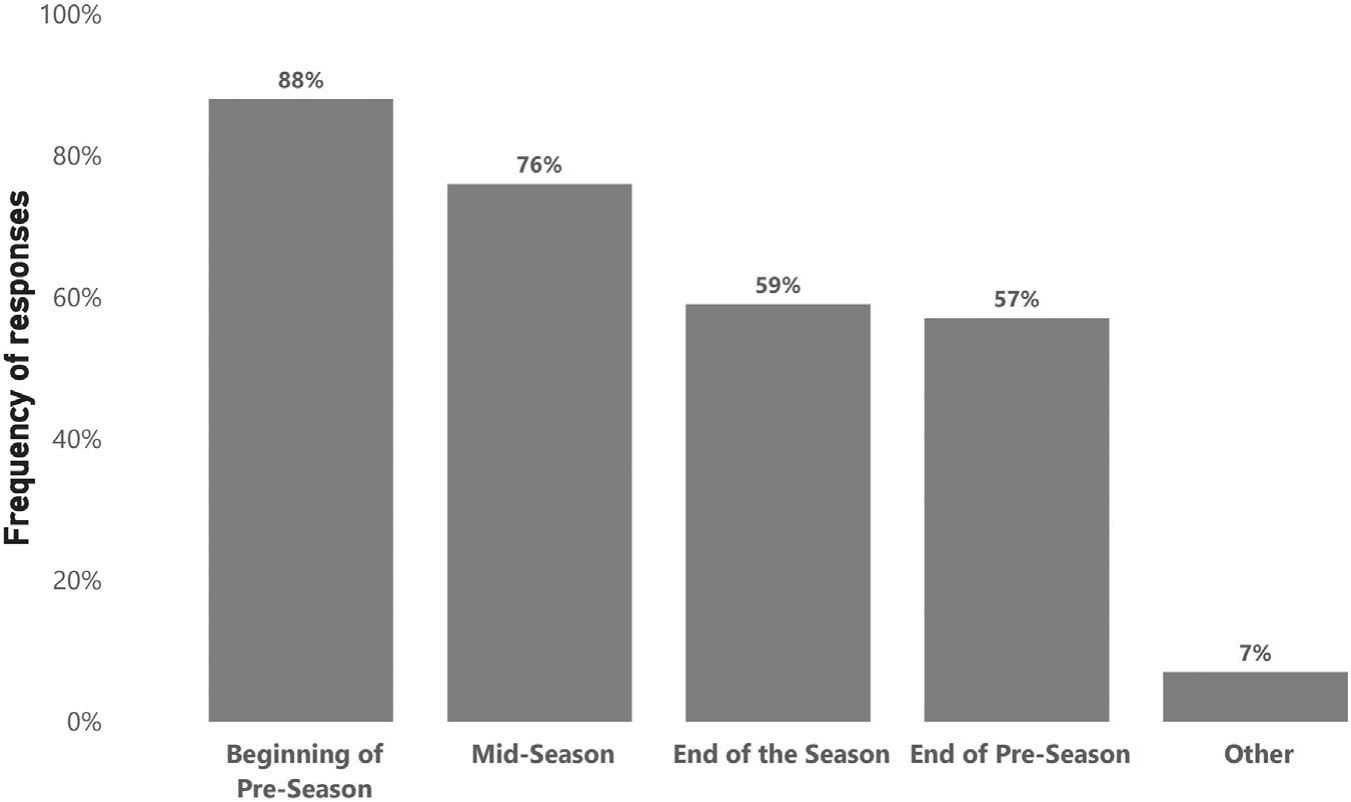
Timing of testing (n = 75).

**FIGURE 3:**
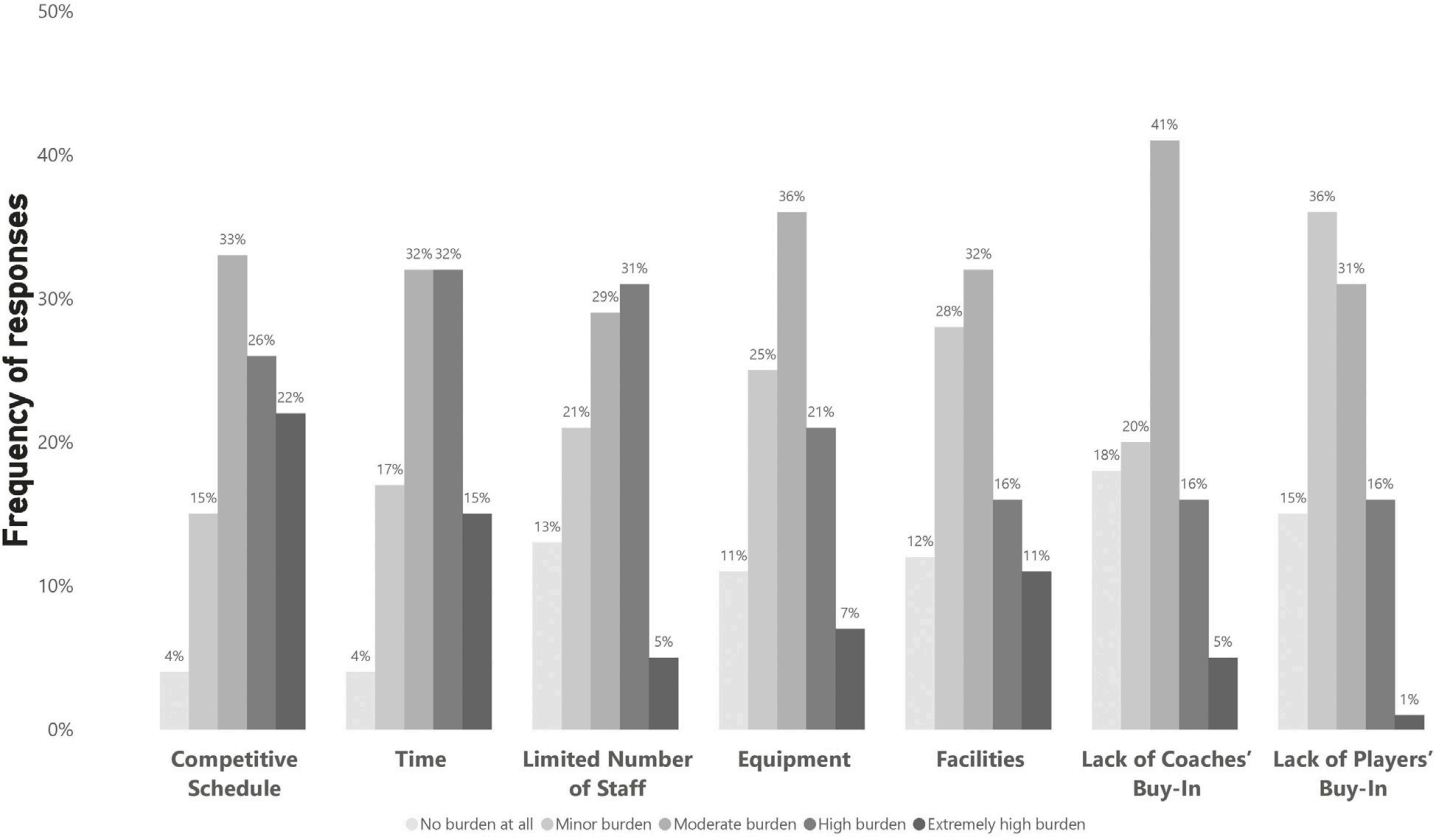
Perceived degree of burden (n = 75).

In total, 75 participants (74%) answered the questions regarding testing implementation. Practitioners reported conducting 3.6 ± 2 formalized fitness testing sessions with their players per season. However, practitioners believed the optimal formalized fitness testing frequency should be 4.5 ± 2 times per season. Figure 2 illustrates when practitioners conduct formalized fitness testing during the season. Figure 3 shows the perceived degree of burden (barriers) in elite male soccer concerning the implementation of fitness testing. In terms of how fitness testing is carried out, 45% reported following a ‘hybrid’ approach, which blended ‘traditional’ (i.e., a day dedicated to testing) and ‘integrated’ (i.e., testing within regular training sessions) methods. Whereas 28% specifically used an ‘integrated approach’ and 27% used a ‘traditional approach’.

### Data analysis

Overall, 73 participants (72%) answered the questions regarding data analysis. Of those, 71% reported using statistical software to analyse fitness testing data, with Microsoft Excel being the most prevalent (95%), followed by R (24%), Google Sheets (22%), SPSS (22%), ‘other’ software (16%) (i.e., Microsoft Power BI, Tableau, and athlete management systems), JASP (9%), and Python (5%). For the analysis of fitness test results, 44% of practitioners use the best and average scores of repeated trials to evaluate performance, while 42% use the best score and 14% use the average score. Regarding the selection of raw or standardized values from fitness testing to analyse and interpret results, 36% of practitioners use raw values, 31% use both, 26% use the method determined by the audience (i.e., coaches or players), and 7% use standardized scores. Table 2 shows the methods practitioners use to interpret fitness test results and determine changes in a player’s performance.

**TABLE 2:** Methods of interpreting fitness test results (n = 73).

Factors	Percentage of respondents
Based on athlete’s previous performance	65/73 (89%)
Based on comparison with normative data or benchmarks (published/squad)	50/73 (68%)
Position-specific comparisons	38/73 (52%)
Taking into account some form of error of the measurement (typical error, minimal detectable change, standard deviation, smallest worthwhile change, confidence intervals)	28/73 (38%)
Based on expert opinions or professional consensus	12/73 (16%)
Other (please specify)	0/73 (0%)

### Data reporting

**FIGURE 4:**
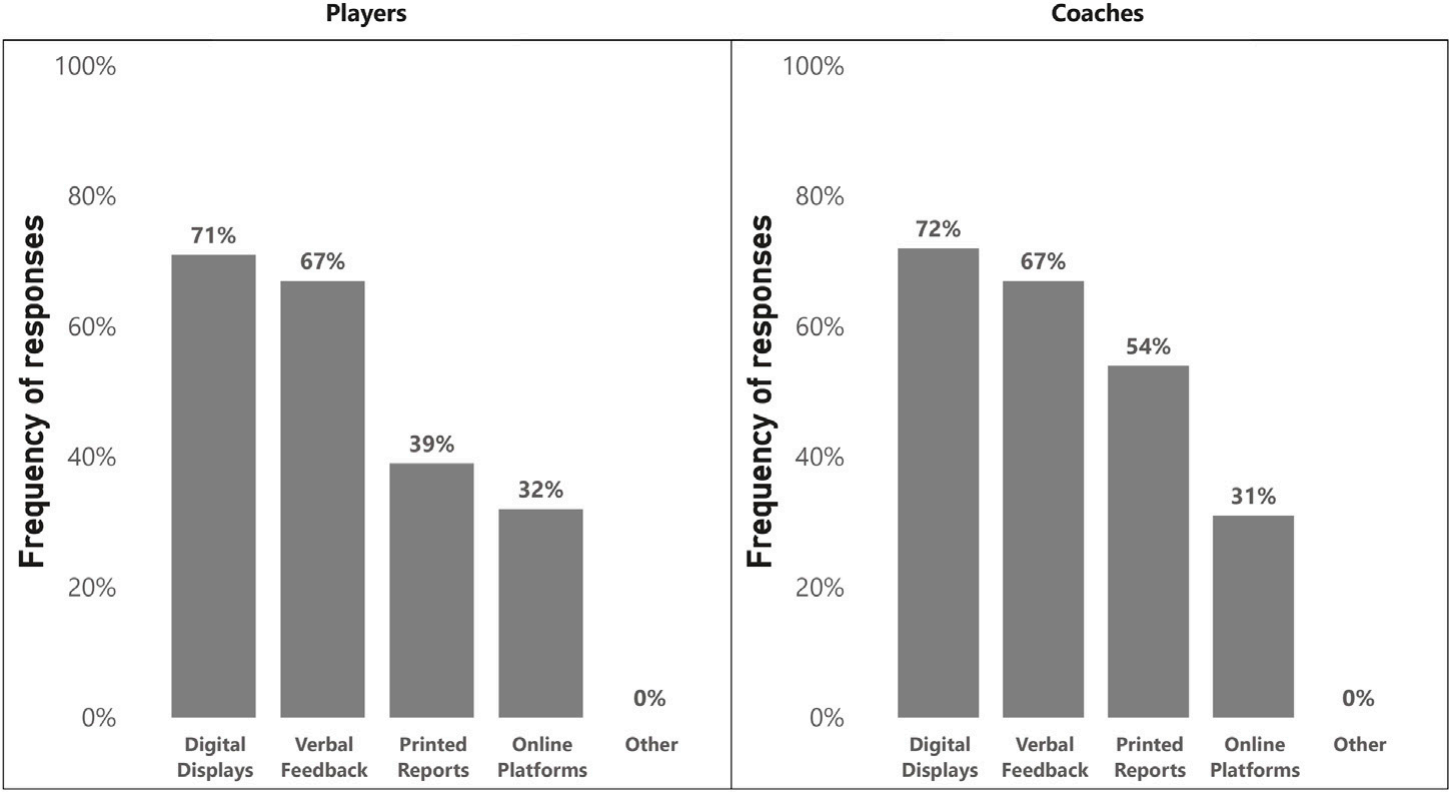
Means of reporting test results (n = 73).

In total, 72 participants (71%) answered the questions regarding data reporting. Most practitioners (96%) reported testing results to their players. Figure 4 illustrates the preferred means for reporting testing results to players and coaches. Microsoft Excel (79%) was the most commonly used software for data visualization, followed by Microsoft Power BI (32%), Google Sheets (21%), ‘other’ software (18%) (i.e., athlete management systems, Microsoft PowerPoint, Prism GraphPad, and Statistica), R (7%), Tableau (7%), Python (4%), and JASP (4%). Interestingly, 55% of practitioners reported using different data visualization methods for coaches and players. Open-ended responses revealed that practitioners generally tailored reports and volume of information to the audience’s needs. For example, players typically received intuitive, individualized reports related to their performance and areas of improvement (i.e., targets for the subsequent testing assessment). Meanwhile, coaches received more comprehensive reports, such as an increased number of variables and more in-depth analyses and comparisons (i.e., team- and position-specific comparisons, use of total score of athleticism).

## Discussion

This study provides insight into the current practices of fitness testing in elite male soccer. To the authors’ knowledge, this is the first study that acquires in-depth information regarding fitness testing selection and implementation in elite soccer across different professional leagues, as well as providing unique insights into the previously unexplored areas of testing results analysis and reporting. The findings of this study can be beneficial for practitioners and researchers working in elite male soccer, illustrating the fitness testing process, analysis and presentation of results, and highlighting areas where standardization may be needed. The discussion will be organized into four sections: a) testing selection, b) testing implementation, c) data analysis, and d) data reporting.

### Testing selection

Strength and aerobic capacity were reported as the most frequently assessed physical capacities in this study, which aligns with a previous survey conducted in elite soccer (Weldon et al., 2021), followed closely by power/reactive strength, and linear speed.

Strength is a fundamental capacity for completing in-game explosive actions, such as sprinting, jumping, and engaging in physical duels (Wing et al., 2020). Moreover, high strength levels can help reduce injury risk (Arnason et al., 2004; Lehance et al., 2008), thereby contributing to increased training and match availability. The isometric mid-thigh pull (IMTP) and adductor squeeze tests are the most commonly performed strength assessments. Both tests present high levels of between-day reliability in elite soccer players, with peak force during the IMTP exhibiting an ICC of 0.88, CV of 5.8%, and SEM of 131 N (Musham and Fitzpatrick, 2020). Furthermore, relative peak torque during the adductor squeeze test showed ICC values ranging from 0.77 to 0.95 and an SEM ranging from 0.08 to 0.18 Nm/kg, depending on the lever length assessed (Light and Thorborg, 2016). The IMTP is an isometric multi-joint test that assesses lower-body strength in a more time-efficient and less fatiguing manner than dynamic testing (e.g., one repetition maximum [RM] back squat) while also providing data and insight into various components of an athlete’s force production ability within a single trial (i.e., peak force, force at specific time points, rate of force development, and impulse) (Brady et al., 2018; Comfort et al., 2019). On the other hand, the adductor squeeze test has been widely implemented due to the role adductor muscles play in soccer-specific tasks, such as kicking, landing, and cutting (Charnock et al., 2009; Rouissi et al., 2016). Also, groin injuries are one of the most affected areas for injury in professional soccer, contributing to 12%–16% of all injuries per season, with an injury incidence of 1.1/1000 h of training and match play (Werner et al., 2009). Previous research has shown that greater isometric adductor strength levels can help reduce injury risk (Moreno-Pérez et al., 2019). In both cases, the growing accessibility of specialized equipment such as force plates and specialized adductor strength testing systems (i.e., ForceFrame, GroinBar, Kangatech KT360) may contribute to their use.

The high prevalence of aerobic capacity testing is unsurprising, considering the high aerobic demands of soccer, where players are required to cover distances up to 14 km per match (Dolci et al., 2020) and the role that aerobic capacity plays in the quick recovery from explosive actions (Stølen et al., 2005). For aerobic capacity assessments, field tests were the most frequently used, such as the 30–15 intermittent fitness test (30–15 IFT) (ICC: 0.80–0.99, CV: 1.5%–6.0%) (Grgic et al., 2021), Yo-yo intermittent recovery test level 1 (YYIR1) (ICC: 0.78–0.98, CV: 4.1%–19.0%) and 2 (YYIR2) (ICC: 0.86–0.96, CV: 4.2%–12.7%) (Grgic et al., 2019), and specified distance time trials. This is unsurprising as field tests are a simple and quick option for practitioners to assess the aerobic capacity of groups of individuals with minimal equipment and preparation (Bok and Foster, 2021).

The occurrence of sprints and jumps preceding some of the most decisive moments of a game, such as scoring a goal (Faude et al., 2012), may explain the high percentage of practitioners that assess power/reactive strength and linear speed capacities. Furthermore, their administration is simple and quick, which allows their integration within gym and field sessions. For power assessments, the countermovement jump (CMJ) (92%) was the most used by practitioners in this study. The CMJ is a time-efficient test that requires minimal athlete familiarization, exhibiting high within- (i.e., ICC: 0.97, CV: 2.7%, SEM: 1.4 cm) and between-day (i.e., ICC: 0.83, CV: 4.3%, SEM: 1.7 cm) reliability in elite soccer players (Enright et al., 2018; Maestroni et al., 2023). Results show that practitioners use a multi-faceted approach to power testing, including other tests such as the squat jump (SJ) (ICC: 0.89, CV: 3.7%, SEM: 1.4 cm) (Enright et al., 2018), single-leg CMJ (SLCMJ) (ICC: 0.70–0.96, CV: 3.7%–13.7%) (Bishop and Read, 2019) and drop jump (DJ) (ICC: 0.95, CV: 2.5%) (Requena et al., 2014), to possibly gain a broader picture of stretch-shortening cycle (SSC) characteristics and inter-limb asymmetry. Concerning linear speed assessment, practitioners generally tested distances <40 m, with 10 m (ICC: 0.78–0.87, CV: 0.8%–3.6%, SEM: 0.02 s) (Bishop and Brashill, 2019; Stern et al., 2020; Bishop et al., 2022), 30 m (ICC: 0.86–0.94, CV: 1.1%–2.3%) (Bishop et al., 2022; 2023), 20 m (ICC: 0.82–0.99, CV: 0.9%–1.3%. SEM: 0.02 s) (Ben Brahim et al., 2021; Bishop et al., 2023), and 5 m (ICC: 0.87–0.99, CV: 1.7%–2.5%, SEM: 0.01–0.02 s) (Bishop and Brashill, 2019; Ben Brahim et al., 2021) sprints being the most commonly selected, in respective order. This selection may be based on match activity profiles, as individual sprints usually last between 2 and 4 s and are typically <20 m in distance (Vigne et al., 2010).

Published scientific literature was reported as the most influential factor for test selection, which could indicate the intention of practitioners to utilize scientifically scrutinized testing methods (i.e., ensuring reliability and validity). The constraints faced in practice (e.g., time, budget, and equipment), expert opinions, and previous professional experience also highly influenced testing selection. These findings support the notion that research may inform practice and practice may inform research. Finally, only one-third of practitioners conducted in-house test-retest reliability to determine their selected tests and metrics, possibly due to time constraints within elite soccer. In-house test-retest reliability helps identify the measurement error, which informs future test selection, selected outcome variables, analysis methods, and interpretation of results (Hopkins, 2000; Turner, 2022). However, an inability to administer in-house reliability analysis can present limitations since within-day and between-day test reliability is not always the same owing to biological variations (Hopkins, 2000; Altmann et al., 2019). Furthermore, the measurement error depends on the familiarity of the athletes with the tests (Comyns et al., 2019), underscoring the importance of assessing the reliability within the specific cohort.

### Testing implementation

Practitioners believed their implemented testing frequency was less than optimal, which suggests practitioners cannot administer fitness testing as frequently or as extensively as desired. The two prominent burdens were time availability and congested competitive schedules, similar to a previous survey in elite soccer (Weldon et al., 2021). Almost half of practitioners adopted a ‘hybrid’ fitness testing approach to overcome this issue. This allows practitioners to combine the benefits of a traditional testing approach (i.e., testing in standalone sessions at specific timepoints) with the continuous monitoring during data collection in regular training sessions, facilitating on-going data-informed decisions.

Regarding timing, fitness testing occurs predominantly at the beginning of the pre-season, which is supported by a previous survey conducted in professional soccer (Weldon et al., 2021). This may be due to time availability as few competitions are held during this period, therefore offering the opportunity to conduct thorough, uninterrupted assessments. Furthermore, fitness testing early in pre-season establishes baseline fitness levels, which lays the foundation for performance goal setting, fatigue monitoring, and return-to-play processes (Maestroni et al., 2023; Weakley et al., 2023). A large proportion of fitness testing was also conducted during mid-season, possibly due to competition breaks, with data being used to assess mid-term progress and inform training adjustments and prescriptions. Fewer practitioners tested at the end of the pre-season period, possibly due to the start of the competitive period, where the main focus is on winning games, and consequently, fitness testing becomes less of a priority. Equally, few practitioners tested during the end of the season as players usually depart for their off-season period immediately after the last game. However, this could inform individualized off-season programming.

### Data analysis

The substantial number of practitioners using statistical analysis or statistical software to analyse fitness testing data supports the notion that practitioners should be proficient with the range of methods by which testing data can be analysed (Turner et al., 2015). Most practitioners used Microsoft Excel, highlighting its role as a fundamental tool for practitioners working in elite soccer. Nevertheless, Microsoft Excel has performance limitations when handling large datasets and has limited advanced statistical analysis capabilities. Consequently, R and SPSS are increasingly utilised beyond academic settings.

Concerning the analysis of fitness testing results, the responses of practitioners indicate the discrepancy and the lack of consensus that exist in the field. Overall, 44% of practitioners use both the best and the mean score of multiple trials, which may suggest that practitioners aim to capture a comprehensive picture of an athlete’s performance. In contrast, 42% reported using only the best score to analyse testing results, which aligns with what is generally performed in research (Claudino et al., 2017). This approach may have limitations since a single trial may not accurately reflect an individual’s overall performance, as previous studies have shown that analysing the results using the best score leads to reduced reliability and sensitivity (Kennedy and Drake, 2021; Howarth et al., 2022). Nevertheless, it would also be prudent to record the best score as the mean score could mask an athlete’s maximal physical capacity by including trials where performance was suboptimal. This highlighted disparity in the methods practitioners use may be an area for future investigation to inform a standardized approach to data analysis in elite soccer.

Differences were also observed for the use of raw or standardized scores in the data analysis and interpretation process. Results demonstrated a similar preference for practitioners to analyse the results based on raw values (36%) and the combination of raw and standardized scores (31%). Raw scores offer the advantage of immediate feedback and direct comparison with an individual’s performance over time. On the other hand, standardized scores express the test results as a standard deviation from the mean, which is valuable to show where the player ranks relative to the group or comparing test performance with different outcome variables (McGuigan et al., 2013; Turner et al., 2019).

Last but not least, some valuable insights can be drawn from the responses on how practitioners interpret fitness testing results. Most practitioners (89%) compared current test results to previous results, which is reasonable as assessing individual changes is the most relevant for the practitioners working in the practical setting to inform the continuation or modification of a training intervention (Ward et al., 2018). Nevertheless, a lower percentage (38%) accounted for measurement error, which allows for the identification of normal variation between testing sessions. This may have major implications for the interpretation of fitness test results, because if a change in performance is not greater than the measurement error, then the change cannot be deemed with confidence as meaningful (Hopkins, 2000). Therefore, this may lead to training interventions being perceived as successful or unsuccessful, and subsequent decisions being ill-informed. Therefore, adopting a more holistic approach to interpreting performance changes may be beneficial. A large percentage (68%) of practitioners compared results with normative data or established benchmarks, which can play a key role in setting performance goals and talent identification (McGuigan et al., 2013). Finally, a position-specific comparison (52%) is performed by practitioners, as different positions have varying physical demands, thus different expected physical profiles (Walker and Hawkins, 2018; Turner et al., 2019).

### Data reporting

Most practitioners (96%) report testing results to the players, demonstrating the importance placed on performance feedback in the athletic development process. Furthermore, informing players of their strengths, weaknesses, and longitudinal progress could increase their engagement and overall buy-in. Digital displays (e.g., static and interactive dashboards) and verbal feedback represent the most prevalent methods of reporting fitness testing results to coaches and players, illustrating a delicate balance between technological use and interpersonal communication. In addition, information from multiple tests can be incorporated into a single document, which is convenient and time-efficient. Nevertheless, verbal feedback remains a critical component of the testing data reporting process since it represents a direct communication method that can convey the nuanced insights and clarifications that a digital display may fail to and provide a basis for discussions about an individual’s progress.

As with data analysis, Microsoft Excel is the most commonly employed software for the visualization of testing results, followed by Microsoft Power BI (32%), which offers more advanced visualization capabilities. Over half (55%) of practitioners differentiated their data visualization strategies for coaches and players. This may indicate the tendency to create tailored data visualization based on the needs of the end audience since the roles of coaches and players are distinct. When delving deeper into those differences, it appears that coaches typically receive more elaborate and in-depth analyses. This increased analysis provides a broader range of information to better assist coaches in their holistic decision-making processes around player development and selection. Conversely, players receive more concise reports primarily focused on their performance, which is key to increasing their awareness of focus areas.

## Limitations and future research

This survey study, although extensive in scope, is not without its limitations. Firstly, a survey cannot encompass all the nuances of fitness testing, and certain components may have been overlooked. Secondly, no comparative analysis was performed between testing practices between first team and youth settings, the objective was to provide an overview of the fitness testing procedures in elite soccer. Future research should examine the nuanced differences between these settings. Thirdly, the lack of transparent definition of the terms “professional” and “elite” may affect the interpretation of the eligibility of the participants in the study (McAuley et al., 2022). For example, in this study, ‘elite’ refers to practitioners working within a professional soccer club with players older than 17 (i.e., first team or youth).

Given the wide range of tests used by practitioners, there is a need for ‘ecologically valid’ (i.e., reflecting and respecting the constraints of the applied elite soccer settings), reliability, and sensitivity studies to determine the practical utility of these tests and their outcome variables. This will inform a simplified testing selection by facilitating an informative and efficient fitness testing process. In addition, future research should investigate the ‘ideal’ approach for analysing fitness testing data to advance the current knowledge in interpreting fitness changes. Last but not least, given the importance of effectively communicating the results from fitness testing, further information on the specific preferences of key stakeholders (i.e., players, coaches, and support staff) in elite soccer should be sought.

## Conclusion

**FIGURE 5:**
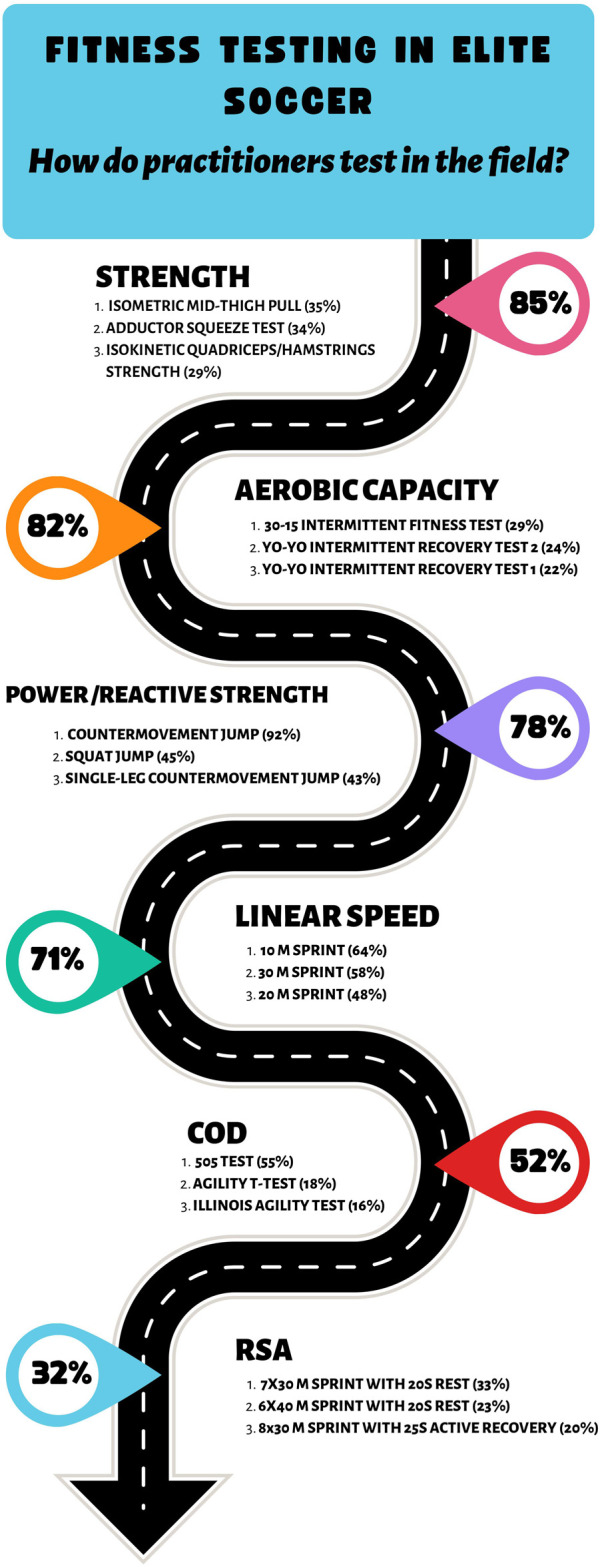
Most commonly assessed physical capacities and tests.

This study presents an in-depth overview of the fitness testing processes in elite male soccer. The infographic in Figure 5 illustrates the most commonly assessed physical abilities and the most commonly administered tests to assess them. Scientific literature is the main influence of test selection, although a pragmatic approach is adopted, as practical constraints and professional experience play an important role. Practitioners tested less frequently than they believed optimal, with time and competitive schedules being the biggest barriers. Consequently, the beginning of the pre-season is the most common time to conduct fitness testing, with competitive periods during the season leaving less time for fitness testing. Therefore, the adoption of ‘hybrid’ fitness testing, whereby standalone testing sessions are concurrently supplemented with integrated testing within training sessions, may help overcome this issue. Microsoft Excel is the most popular software amongst practitioners for testing data analysis and visualization. A similar number of practitioners use either the combination of the mean and the best score or the best score in results analysis, possibly indicating a need for a standardized approach. Comparing a player’s test performance with previous scores was the most commonly reported method for interpreting test results. However, a substantially lower percentage utilizes some form of error measurement. Digital displays and verbal feedback are the most commonly used data reporting methods. A tendency towards tailored visualizations for coaches and players was identified, with the main difference being that coaches may receive a greater depth of information than players.

## Data Availability

The original contributions presented in the study are included in the article/Supplementary Material, further inquiries can be directed to the corresponding author.
