# Supplementary material for: A survey into the current fitness testing practices of elite male soccer practitioners: from assessment to communicating results
Source: Front Physiol. 2024 Mar 19;15:1376047. doi: 10.3389/fphys.2024.1376047 (PMC10985349; doi:10.3389/fphys.2024.1376047)
Supplement: Supplementary file 1 [file Table1.DOCX]

**APPENDIX 1.**

**SURVEY: A Survey into the Current Fitness Testing Practices of Elite Soccer Practitioners: From Assessment to Communicating Results**

**■** Refers to questions with multiple choice answers

**●** Refers to questions with single choice answers

Other: Was provided for a number of questions in order for participants to provide specific answers, if their practices are different to the pre-determined answers, or if they wished to further elaborate on their answers.

1. **Background Information**

Q1. What is your current role?

- Director of Performance
- Sport Scientist
- S&C Coach
- Physical Performance Coach
- Rehabilitation Coach/Athletic Trainer
- Football Coach
- Physiotherapist
- Medical Doctor
- Other (please specify)

Q2. In which country do you currently work as a practitioner?

Q3. How many years of experience do you have working as a practitioner in elite-level soccer?

Q4. What is your academic degree level?

Q5. Do you possess any of the following certifications?

- Certified Strength and Conditioning Specialist (CSCS) from NSCA
- Certified Performance and Sport Scientist (CPSS) from NSCA
- Accredited Strength and Conditioning Coach (ASCC) from UKSCA
- BASES Accreditation
- Coaching Licence (UEFA, The FA, or your own national soccer association)

1. **Testing Selection**

Q6. Which of the following physical capacities do you test in your elite soccer program?

- Strength
- Linear Speed
- Change of Direction (COD)
- Repeated Sprint Ability (RSA)
- Power/Reactive Strength
- Aerobic Capacity

Q7. Which fitness tests do you use to assess aerobic capacity? (Eligibility to answer if aerobic capacity was selected as an assessed physical capacity)

- Yo-yo Intermittent Recovery Test 1
- Yo-yo Intermittent Recovery Test 2
- 30-15 Intermittent Fitness Test
- University of Montreal track test (UM-TT)
- Incremental treadmill test to exhaustion
- VAMEVAL Test
- Submaximal Test
- Multi-stage Fitness Test (Also known as beep test,bleep test, 20 m Shuttle Run Test)
- Specified Distance for Time
- Specified Time for Distance
- Other (Please Specify)

Q8. Which fitness tests do you use to assess linear speed? (Eligibility to answer if linear speed was selected as an assessed physical capacity)

- 5 m
- 5 m Flying
- 10 m
- 10 m Flying
- 20 m
- 20 m Flying
- 30 m
- 30 m Flying
- 40 m
- 40 m Flying
- Other (Please Specify)

Q9. Which fitness tests do you use to assess change of direction ability? (Eligibility to answer if change of direction ability was selected as an assessed physical capacity)

- 505 COD Test
- Agility T-Test
- Zig-Zag Test
- Illinois Agility Test
- Arrowhead Agility Test
- Other (Please Specify)

Q10. Which fitness tests do you use to assess repeated sprint ability? (Eligibility to answer if repeated sprint ability was selected as an assessed physical capacity)

- 7 x 30m Sprint with 20 s Rest
- 6 x 40m Sprint with 20 s Rest
- 8 x 30 m Sprint with 25 s Active Rest
- 6 x 40 m (20 + 20 m with 180° turns) Shuttle Sprints separated by 20 s of Passive Rest
- Other (Please Specify)

Q11. Which fitness tests do you use to assess strength? (Eligibility to answer if strength was selected as an assessed physical capacity)

- Isometric Mid-Thigh Pull
- Isokinetic strength of the Quadriceps and/or Hamstrings
- 1RM Back Squat
- 1RM Bench Press
- 3RM Squat
- 3RM Bench Press
- Predicted 1RM using Barbell Velocity
- Isometric Adductor Strength (Adductor Squeeze Test)
- Isometric Squat
- Flywheel Testing
- Other (Please Specify)

Q12. Which fitness tests do you use to assess power/reactive strength? (Eligibility to answer if power/reactive strength was selected as an assessed physical capacity)

- Countermovement Jump
- Single-leg Countermovement Jump
- Squat Jump
- Drop Jump
- Unilateral Drop Jump
- Vertical Jump (Free Arms)
- Single-leg Hop Test
- Triple Hop Test
- 10/5 Repeated Jumps Test
- Other (Please specify)

Q13. How do you determine which fitness tests to use in your program?

- Published scientific literature
- Expert opinion or professional experience
- Constraints (e.g., time, budget, equipment)
- Specific needs or goals of the team or players
- Needs analysis of the sport
- Prescribed from/used on national governing bodies
- Usefulness of a test (established from in-house test-retest reliability)
- Other (Please Specify)

1. **Testing Implementation**

Q14. How many times per season do you conduct formalised fitness testing with your players?

Q15. How many times per season do you think formalised fitness testing should be conducted for soccer players?

Q16. When do you conduct fitness testing during the season?

- Beginning of pre-season
- End of pre-season
- Mid-season
- End of the season
- Other (Please Specify)

Q17. How do you conduct fitness testing in the setting that you currently work?

- Formalised/Traditional approach (i.e., a day dedicated to testing)
- Integrated approach (i.e., testing conducted within the regular testing sessions)
- Hybrid approach (Combination of both)

Q18. Please rate the degree of burden that each of the following barriers places on conducting fitness testing in your setting (1 = No burden at all, 2 = Minor burden, 3 = Moderate burden, 4 = High burden, 5 = Extremely high burden)

- Time
- Equipment
- Facilities
- Lack of coaches’ buy-in
- Lack of players’ buy-in
- Limited number of staff
- Competitive schedule

1. **Data Analysis**

Q19. Do you use any form of statistical analysis or statistical software to analyse the fitness testing data in your elite soccer environment?

- Yes
- No

Q20. Which of the following software do you use? (Eligibility to answer if “Yes” was selected as a response in Q19)

- Microsoft Excel
- Google Sheets
- Python
- R
- SPSS
- JASP
- Other (Please Specify)

Q21. Do you use the mean value of the repeated trials or the best value for the analysis of the results?

- Mean value
- Best value
- Both

Q22. How do you interpret the results of the fitness tests in your elite soccer program?

- Based on comparison with normative data or benchmarks (published/squad)
- Based on athlete’s previous performance
- Based on expert opinions or professional consensus
- Position-specific comparisons
- Taking into account some form of error of the measurement (Typical Error, Minimal Detectable Change, Standard Deviation, Smallest Worthwhile Change, Confidence Intervals)
- Other (Please Specify)

Q23. Do you interpret the results based on the raw values of the fitness test result data or do you convert them into a standardised form (z-score, t- score, Standard Ten [STEN] scores, percentiles)?

- Raw values
- Standardized form
- Both
- Depends on the end audience (i.e., coaches, players)

1. **Data Reporting**

Q24. Do you report the test results to your players?

- Yes
- No

Q25. How are fitness test results typically reported to the players in your elite soccer program?

- Printed reports
- Digital displays
- Online platforms
- Verbal feedback
- Other (Please specify)

Q26. How are fitness test results typically reported to the coaches in your elite soccer program?

- Printed reports
- Digital displays
- Online platforms
- Verbal feedback
- We don’t report them at all
- Other (please specify)

Q27. Which of the following software do you use to visualize the fitness test results?

- Microsoft Excel
- Google Sheets
- Python
- R
- Power BI
- Tableau
- JASP
- Other (Please Specify)
- None of the above

Q28. Do the methods of data visualization (i.e. graphs, metrics and information provided) differ between players and coaches?

- Yes
- No

Q29. How do the methods of data visualization (i.e. graphs, metrics and information provided) differ between players and coaches? (Eligibility to answer if “Yes” was selected as a response in Q28)
